# The origin of anticorrelation for photon bunching on a beam splitter

**DOI:** 10.1038/s41598-020-64441-2

**Published:** 2020-04-30

**Authors:** Byoung S. Ham

**Affiliations:** Center for Photon Information Processing, School of Electrical Engineering and Computer Science, Gwangju Institute of Science and Technology, Gwangju, South Korea

**Keywords:** Optics and photonics, Physics

## Abstract

The Copenhagen interpretation, in which the core concepts are Heisenberg’s uncertainty principle and nonlocal EPR correlation, has been long discussed. Second-order anticorrelation in a beam splitter represents the origin of these phenomena and cannot be achieved classically. Here, the anticorrelation of nonclassicality in a beam splitter is interpreted using the concept of coherence. Unlike the common understanding of photons having a particle nature, anticorrelation is rooted in the wave nature of coherence optics, described by coherence optics, wherein quantum superposition between two input fields plays a key role. This interpretation may pose fundamental questions about the nature of nonclassicality and pave a road to coherence-based quantum information.

## Introduction

In general, the Copenhagen interpretation focuses on the contradictory concept of nonlocal correlation represented by EPR^1^. This nonlocal correlation between two remote objects beyond the physical reality (or local realism) has become the foundation of quantum information processing^2^. The major benchmark for EPR is Bell’s inequality^3^ for a mathematical form or the CHSH inequality^4^ for a physical form, in which inequality is violated only by nonclassical quantum mechanics. However, nonlocal EPR correlation is based on coherence and proved by destructive quantum interference-based anticorrelation in a beam splitter (BS)^5^, where nonlocal objects are strictly phase-dependent^5,6^. Thus, the definition of classicality in the Copenhagen interpretation must be confined to incoherence optics which represents independent and individual objects with no phase relation^3–5^. This viewpoint makes the present paper unique and enriches the conceptual foundation of EPR as well as quantum information.

In both quantum^2^ and classical^7^ information processing, quantum superposition plays a key role. A typical example of quantum superposition is Young’s double-slit experiment which is effective for both coherent fields (wave nature)^8^ and single photons (particle nature)^9–11^, resulting in first-order correlation, g^(1)^. Regardless of the input characteristics, however, the resulting fringe is due to coherence between the two paths. Thus, Young’s double slit can be replaced by a 50:50 nonpolarizing BS. The BS-based Young’s double slit experiment has also been performed in a Mach-Zehnder interferometer (MZI) with one input^11^. If two input fields are applied to the BS, then the second-order correlation ${g}^{(2)}$ is related to the two outputs with each output a superposed form of both inputs^5^. The sub-Poisson photon statistics of the anti-correlation in ${g}^{(2)}$ indicates that the input photons are anti-bunched nonclassical particles^10^. Here, we present that anticorrelation is a direct result of coherence optics between two input photons with a particular phase relationship. Furthermore, anticorrelation in a BS can also be achieved using an MZI, and its output superposition results in entanglement superposition.

The behavior of ${g}^{(2)}$ relies on the input fields’ characteristics^12^: super-Poisson (or ${g}^{(2)} > 1$) for thermal or chaotic light; Poisson (or ${g}^{(2)}=1$) for coherent light; sub-Poisson (or ${g}^{(2)} < 1$) for anti-bunched photons. Hanbury Brown and Twiss (HBT) first applied super-Poisson input fields to high-resolution spectroscopy of distant stars in 1956^13^, where the enhanced effect is due to intensity correlation added to the incoherence background $({g}^{(2)}=1)$. The opposite case wherein ${g}^{(2)} < 1$ was first observed by Hong-Ou-Mandel (HOM) using entangled photon pairs generated by a spontaneous parametric down conversion (SPDC) process in 1987^5^. This weird phenomenon of perfect anticorrelation is due to photon bunching into one of two output paths. On the contrary, coherent light input have been understood to follow Poisson statistics $({g}^{(2)}=1)$, limiting the HOM dip.

In the area of quantum information, the photon bunching phenomenon in a beam splitter has been intensively studied in pursuit of a basic understanding of nonclassical physics using antibunched single photons^14–22^. According to Heisenberg’s uncertainty principle, a single photon can be described as a fixed energy object with no particular phase, comparable to an unlocalized electron in Bohr’s atomic model: Copenhagen interpretation. The bunching of single photons seen in the HOM experiments seems at first glance to be a result of incoherence because of its random phase. However, this is incorrect because the lower bound of ${g}^{(2)}$ for incoherent light is, at most, 1/2 (see the Supplementary Information A)^19,21,22^. Although a lot of studies have been performed for BS-based ${g}^{(2)}$ correlation, it is not yet clearly understood what causes anticorrelation, ${g}^{(2)}(\tau =0)=0$. Here, the photon bunching observed in HOM experiments is newly interpreted as being a special case of coherence optics, resulting in sub-Poisson statistics of a nonclassical nature. This results, which seemingly conflict with conventional photon statistics, poses a fundamental question of what nonclassicality should be. The answer to this question may lead us to a better understanding of quantum optics and open the door to quantum superposition-based applications such as unconditionally secured classical key distributions^23^ and superposition-enhanced machine learning^24^.

## Results

**Figure 1:**
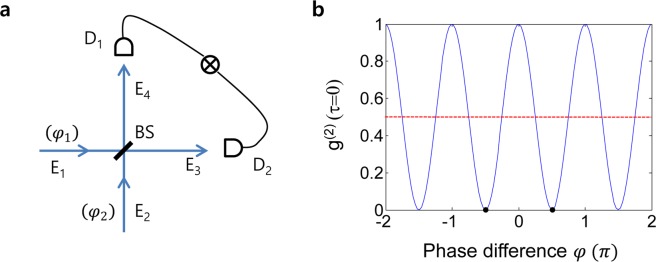
Intensity correlation. (**a**) A schematic of second-order correlation measurements. (**b**) Numerical simulation of ${g}^{(2)}(\tau =0)$ when E_1_ and E_2_ are coherent with a $\frac{\pi }{2}$ phase difference: Red-dotted line is the lower bound of incoherence optics. BS, beam splitter; E_i_, Electric field; D_i_, photon detector. $\varphi ={\varphi }_{1}-{\varphi }_{2}$. Two dots are for $\varphi =\pm \frac{\pi }{2}$.

The BS matrix, [BS], has been clearly analyzed for the split output fields (E_3_ and E_4_) with respect to the input fields (E_1_ and E_2_) in 1980 (see Fig. 1)^25^. For each input field, [BS] is described by^25^:

1 $$[BS]=\frac{1}{\sqrt{2}}[\begin{array}{cc}1 & i\\ i & 1\end{array}],$$

where the imaginary number *i* stands for a π/2 phase shift between two coherent outputs. Thus, the output fields in Fig. 1(a) can be described in a simple matrix form:

2 $$[\begin{array}{c}{E}_{3}\\ {E}_{4}\end{array}]=[BS][\begin{array}{c}{E}_{1}\\ {E}_{2}\end{array}].$$

To study the second-order correlation in a classical regime of coherence optics, the inputs are set to be traveling light fields with different wavelengths and different initial phases for generality:

$${E}_{1}={E}_{0}{e}^{i({k}_{1}r-{w}_{1}t+{\varphi }_{1})};{E}_{2}={E}_{0}{e}^{i({k}_{2}r-{w}_{2}t+{\varphi }_{2})},$$

where ${k}_{i}$, ${w}_{i}$, ${\varphi }_{i}$ are wave vector, angular frequency, and initial phase of each field, respectively. The coincidence detection for two outputs (E_3_ and E_4_) is represented by the second-order correlation function, ${g}^{(2)}$:

3 $${g}^{(2)}(\tau )=\frac{\langle {E}_{3}^{\ast }(t){E}_{3}(t){E}_{4}(t+\tau ){E}_{4}^{\ast }(t+\tau )\rangle }{\langle {E}_{3}^{\ast }(t){E}_{3}(t)\rangle \langle {E}_{4}^{\ast }(t){E}_{4}(t)\rangle },$$

where τ is the temporal delay between two outputs at detectors. Here, it should be noted that the anticorrelation is satisfied by both coincidence arrival of two input photons on a BS to satisfy destructive quantum interference. The two split outputs (E_3_ and E_4_) from one input (E_1_ or E_2_) by a BS in Fig. 1(a) are automatically coherent each other regardless of the bandwidth, photon numbers, and phase fluctuation of the input field. Even for a single photon with a random phase, the split outputs are coherent each other in a form of superposition. This is the physical origin of self-interference for a single photon- (or wave-) based Young’s double-slit experiment. Thus, the BS physics is consistent regardless of the photon characteristics whether it is a single photon or a wave. The HBT is also satisfied for the inputs of chaotic light satisfying the BS matrix (see the Supplementary Information B)^13^. Here, the chaotic light is defined by photon statistics of blackbody radiation or thermal lights, where ${g}^{(2)} > 1$ is due bunched incoherent photons^26^. The HOM dip is for two coherent input photons as described in Fig. 1(a) satisfying the BS matrix, too (discussed in *Analysis*).

Based on two independent input fields of E_1_ and E_2_ in Fig. 1(a), each output can be described as coherent superposition of the inputs from Eq. (2):

4 $$\begin{array}{c}{E}_{3}=\frac{1}{\sqrt{2}}({E}_{1}+i{E}_{2}),\\ \,=\frac{1}{\sqrt{2}}{E}_{0}({e}^{i({k}_{1}r-{w}_{1}t+{\varphi }_{1})}+i{e}^{i({k}_{2}r-{w}_{2}t+{\varphi }_{2})})\end{array}$$

5 $$\begin{array}{c}{E}_{4}=\frac{1}{\sqrt{2}}(i{E}_{1}+{E}_{2})\\ \,=\frac{1}{\sqrt{2}}{E}_{0}(i{e}^{i({k}_{1}r-{w}_{1}t+{\varphi }_{1})}+{e}^{i({k}_{2}r-{w}_{2}t+{\varphi }_{2})})\end{array}$$

According to the definition of the second-order intensity correlation $\,{g}^{(2)}$, Eq. (3) at coincidence detection results in (see the Supplementary information A):

6 $${g}^{(2)}(0)=\frac{1}{2}\langle 1+cos2(\Delta +\varphi )\rangle ,$$

where $\Delta =({k}_{1}-{k}_{2})r-({w}_{1}-{w}_{2})t$. The relative phase, $\varphi ={\varphi }_{1}-{\varphi }_{2}$, is now fixed at a particular value regardless of time variation of the fields. From Eq. (6), the solution for anticorrelation $({g}^{(2)}(0)=0)$ is obtained as:

7 $$\Delta +\varphi =\pm \left(n-\frac{1}{2}\right)\pi ,\,n=1,2,3\ldots $$

Because the path lengths are fixed for coincidence detection in Fig. 1(a), the frequency difference-caused phase fluctuation in Δ dominates ${g}^{(2)}(0)$ if two inputs are not degenerate $({w}_{1}\ne {w}_{2})$.

Now, we analyze Eq. (6) for two different categories determined by the input field’s phase relationship: Coherence vs. Incoherence. We assume that the input fields are monochromatic for simplicity. The delay time τ between two outputs at both detectors is set to be zero to satisfy the coincidence detection. Furthermore, a typical HOM dip is analyzed for the proof of concept.

## Analysis

### **Coherence optics for**${{\boldsymbol{g}}}^{(2)}$

For the same-wavelength input fields E_1_ and E_2_ $(\Delta =0,\,\varphi \ne 0)$ in Fig. 1(a), Eq. (6) results in a $\varphi -\,$dependent ${g}^{(2)}$. In this case, the two input fields cannot be discernible by the BS or detectors, regardless of $\varphi$, satisfying the indistinguishability condition in HOM experiments^5^. This indistinguishability originates in Young’s double-slit experiment using single photons^11^. Here, the input field’s bandwidth determines the coherence time of ${g}^{(2)}$ for coincidence detection after the BS^5^. Figure 1(b) shows the numerical calculations for the second-order correlation ${g}^{(2)}$ as a function of $\varphi$ at ${\rm{\tau }}=0$. This ${g}^{(2)}$ modulation is phase sensitive to the two input fields (photons) of the BS, which has never been discussed before although it has been observed^6^. If two input fields are in phase $(\varphi =0)$ or out of phase $(\varphi =\pi )$, then the Poisson statistics of the coherence feature is satisfied for ${g}^{(2)}=1$. If there is a $\pm {\rm{\pi }}/2$ phase shift between the two input fields, then perfect anticorrelation of the nonclassical features results in ${g}^{(2)}(\tau =0)=0$ (see the two dots in Fig. 1(b)). This anticorrelation at ${\varphi }_{n}=\pm \left(n-\frac{1}{2}\right)\pi$ obviously conflicts with the conventional understanding of ${g}^{(2)}$ correlation based on the particle nature of photons^5^. The “n” in ${\varphi }_{n}$ denotes bases in a Hilbert space (discussed later). The interesting result is that Eq. (7) applies only for the inputs before the BS. Depending on the sign selection of ${\varphi }_{n}$ in Eq. (7), the output channel selection is deterministic for photon bunching (see the Supplementary Information A). By combining the symmetric phase pair of ${\varphi }_{n}$, e.g., ${\varphi }_{1}=\pm \frac{\pi }{2}$, no which-way information for the output paths (or photons) is satisfied via superposition of the anti-correlation bases. Briefly we concluded that the physical origin of anticorrelation in a BS is not due to detection of independent single photons but is due to the destructive quantum interference between two input modes satisfying Eq. (7). The missing parameter ${\varphi }_{n}$ in the input mode is the most important discovery in the present study, and has never been discussed before. Moreover, any photon pair used for a HOM dip experiment should be pre-determined to have a fixed phase relation, otherwise it will be governed by incoherence optics (discussed later).

### **Incoherence optics for**${{\boldsymbol{g}}}^{(2)}$

**Figure 2:**
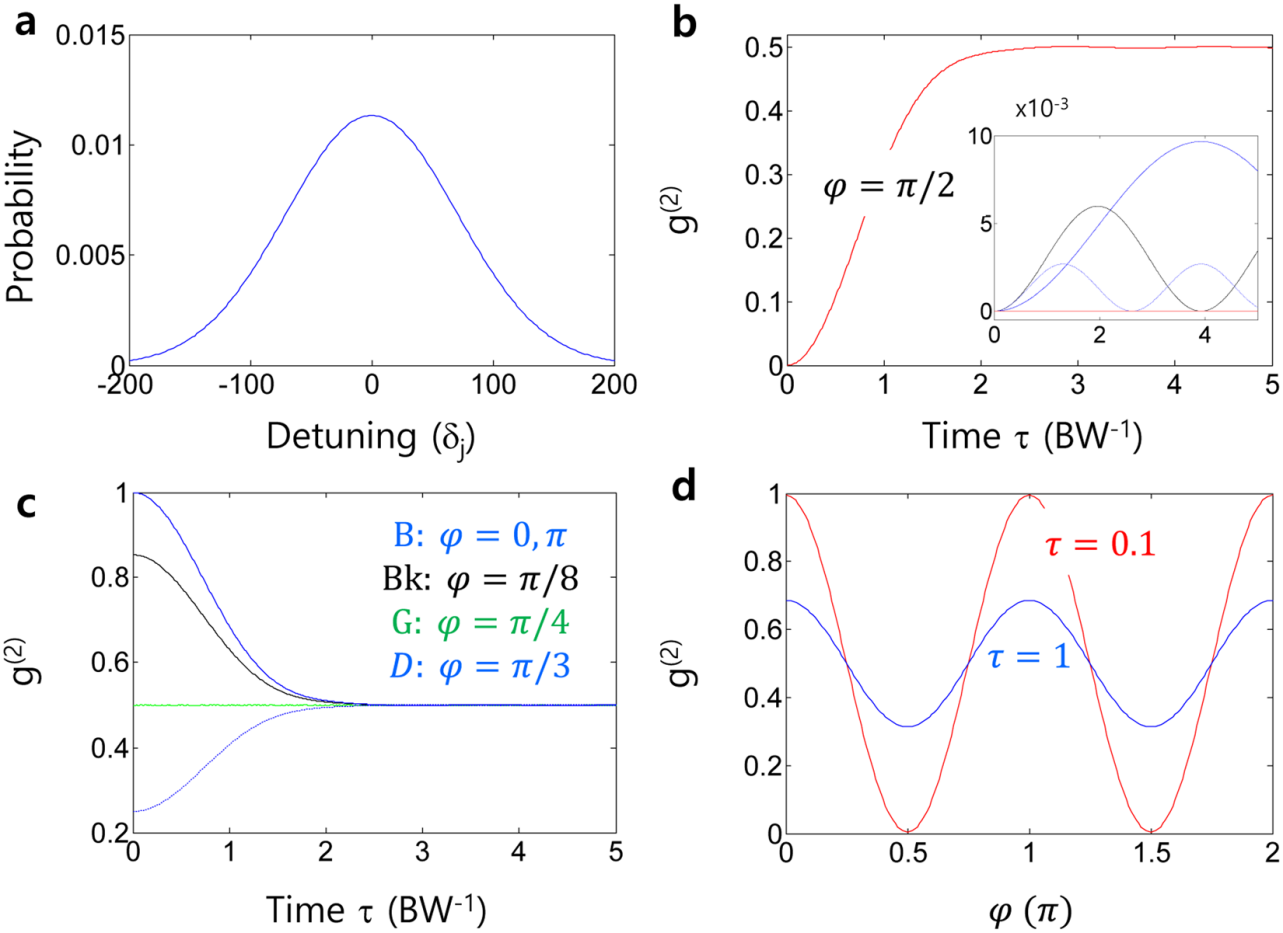
Numerical analyses for a HOM dip. (**a**) Photon bandwidth in SPDC-HOM. BW=100 GHz. (**b**) Sum of individual photons randomly distributed in (**a**) for $\varphi =\pi /2$. The inset shows individual signals: R, ${\delta }_{j}=0$; B, ${\delta }_{j}=40$; Bk, ${\delta }_{j}=80$; Dotted, ${\delta }_{j}=120$ GHz. (**c**) For different ${\rm{\varphi }}$ in (**b**). (**d**) The ${\rm{\varphi }}$-dependent g^(2)^ for different τ (BW^−1^). The calculations are based on Eq. (6) with a detuning step at 2 GHz. The τ affects only the frequency difference in Δ of Eq. (7).

If the two input fields E_1_ and E_2_ in Fig. 1(a) are independent of each other with random phase fluctuations, the parameter $\Delta$ in Eq. (6) plays a key role. The time average of its cosine function becomes zero, resulting in ${g}^{(2)}(0)=1/2$, regardless of $\varphi$ (see the red-dotted line in Fig. 1(b)). Although this value indicates sub-Poisson photon statistics, it is actually the lower bound of classical physics in incoherence optics^19^. However, violation of the lower bound of incoherence optics for ${g}^{(2)}(\tau =0) < 0.5$ has also been observed in nondegenerate HOM experiments^22,27–29^. This unexpected observation is not due to the violation of incoherence optics but results from the coherence optics of HOM experiments via phase matching as well as quantum beating between the two nondegenerate SPDC photons (analyzed in Fig. 2).

### A HOM dip

To understand ${g}^{(2)}$ violation in incoherence optics^28,29^, let us look at a typical SPDC process for a HOM dip with degenerate down-converted photon pairs. Figure 2 shows the result of Eq. (6) satisfying Eq. (7) for randomly detuned photon pairs. Figure 2(a) represents the spectral distribution of the SPDC-generated photons as a Gaussian function with bandwidth BW. The value of δ_j_ is measured in GHz but is meaningful with respect to decoherence time τ (BW^−1^). Although it appears the same in ref. ^5^ due to the simultaneous path length control for both input and output photons, this time τ is actually different from the delay time τ in g^(2)^ in Eq. (3), in which no delay is assumed in Fig. 2. Due to the wide bandwidth $({\rm{BW}}= \sim \,10\,{\rm{nm}})$ of SPDC-based photon pairs, the HOM dip should satisfy the nondegenerate case of incoherence optics regardless of the pumping method^5,6,14–22,27–29^. However, each down-converted photon pair is always phase matched via ${\chi }^{(2)}-$ SPDC nonlinear process at coincidence detection, ${{\boldsymbol{k}}}_{P}={{\boldsymbol{k}}}_{S}+{{\boldsymbol{k}}}_{I}$ and ${\omega }_{P}={\omega }_{S}+{\omega }_{I}$, where ${{\boldsymbol{k}}}_{j}$ and ${\omega }_{j}$ are, respectively, the wave vector and angular frequency of photon j. The subscripts of P, S, and I stand for the pump, signal, and idler photons, respectively. Due to the double spontaneous emission process of ${\chi }^{(2)}$, the phase $\varphi$ between the photon pair should be ${\rm{\pi }}/2$ to compensate for the pump field-excited π phase shift, inherently satisfying Eq. (7). Such a π/2 phase relation between entangled photon pairs has already been proved in independently trapped ions^30^. In most HOM experiments, the average number of photons counted by a photon detector is less than a million per second. Considering the ${g}^{(2)}$ processing and detection time is on the order of nanoseconds, there is no more than one photon pair per event contributing to the ${g}^{(2)}$ value. Furthermore, each event contributes independently to the ${g}^{(2)}$ value regardless of their detuning, δ_j_^29^. Here, the weight factor of each event is determined as a probability solely by the detuning δ_j_. In other words, the different phases initially given to each SPDC photon pair do not matter to the ${g}^{(2)}$ value because of their fixed relative phase ${\rm{\varphi }}$. Thus, the overall ${g}^{(2)}$ is δ_j_-dependent resulting in a BW^−1^ (or τ)−dependent decay as shown in Fig. 2(b). If there is more than one photon per event, then the ${g}^{(2)}$ value should also deteriorate due to δ_j_-caused interference.

Figure 2(b) shows the overall ${g}^{(2)}$ for the 201 δ_j_-dependent events in Fig. 2(a) satisfying Eq. (7) for the anticorrelation condition of $\varphi =\pi /2$. The ${g}^{(2)}$ decay in Fig. 2(b) represents the decoherence due to the δ_j_ effect on bandwidth (BW), where the spatial time delay between the BS and the detectors is governed by ***k***_***j***_ vectors. Even beyond the coherence time, the overall ${g}^{(2)}$ converges to the lower bound of incoherence optics as expected because the actual time delay between the two output photons is not considered in Fig. 2. This is because the $\varphi -\,$dependent ${g}^{(2)}$ oscillation period is much shorter than the HOM spatial bandwidth^5,6^. The inset of Fig. 2(b) shows individual δ_j_-dependent ${g}^{(2)}$ evolutions, where the oscillation period relies on δ_j_. If Eq. (7) is violated, then ${g}^{(2)}$ shows a huge fluctuation depending on $\varphi$ as shown in Fig. 2(c) (see also Fig. 1(b)). Figure 2(d) shows the oscillation in ${g}^{(2)}$ as a function of the difference phase $\varphi$ (see Fig. 1)^6^. Here, it should be noted that Eq. (6) applies for both degenerate^5,6^ and nondegenerate^28,29^ SPDC processes. Due to the wide bandwidth of SPDC, the degenerate case has no practical advantage as discussed above if the detection time is fast enough. For the nondegenerate case, a beating signal between two different center-frequencies is added, resulting in a narrow spatial bandwidth for the anticorrelation^29^. Thus, the violation of ${g}^{(2)} < 1/2$ for the seemingly incoherence-optics-based nondegenerate SPDC-HOM experiments is explained by Fig. 2 using a simple bandwidth model for coherence optics^28,29^.

As analyzed above, the anti-correlation or photon bunching phenomenon for ${g}^{(2)}(\tau =0)=0$ is caused by destructive quantum interference between two input fields (photons) at a particular phase, where each output field is a superposition state of the inputs. Furthermore, this destructive quantum interference induces the missing parameter ${\varphi }_{n}$ in Eq. (7). The anticorrelation seen in HOM experiments has also been attributed to wide bandwidth photon pairs with the same phase difference in Fig. 2 and satisfying Eq. (6). The input photon phase relation satisfying Eq. (7) is much more sensitive than coincidence timing at detectors^5,6^, where the intensity correlation ${g}^{(2)}$ rapidly oscillates as shown in Figs. 1 and 2(d). As experimentally demonstrated^6^, the modulation period of ${g}^{(2)}$ exactly matches the wavelength of the SPDC ${\chi }^{(2)}$ generated input photons, satisfying the physics of Eq. (6). Regarding destructive quantum interference, thus, it can be intuitively understood that an additional π/2-phase shift in Eq. (7) works together with a BS induced π/2-phase shift in Eq. (1). Equation (7) also supports the phase relation between the down-converted photons in the SPDC process, where the π/2-phase shift compensates for the pump field-induced π-phase shift caused by excited atoms.

**Figure 3:**
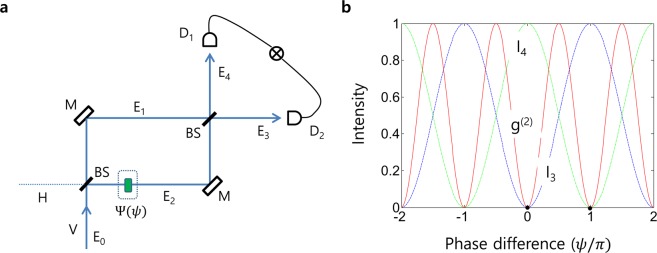
A Mach-Zehnder interferometer for intensity correlation. (**a**) A schematic of a coherence-optics-based HOM setup. BS, beam splitter; E_i_, i^th^ coherent light field; D_i_, i^th^ photodetector; M, mirror. (**b**) Numerical calculations for (a) at ${\rm{\tau }}=0$. Red: ${g}^{(2)}$ correlation (normalized). The blue (green dash-dot) dashed curve is for I_3_ (I_4_). With $\psi =\pm \,2n\pi$ the output is bunched into E_4_. With $\psi =\pm \,(2n-1)\pi$ it goes to E_3_.

Now, our interest is in the missing parameter and its symmetric property: $\,{\varphi }_{n}=\pm \left(n-\frac{1}{2}\right)\pi$. If a particular sign of ${\varphi }_{n}$, say ${\varphi }_{+1}=+\frac{\pi }{2}$, is assigned to E_2_ in Fig. 1(a), then the bunched photon goes for E_4_ according to Eq. (2). This input phase-dependent output determinacy is shown in Fig. 3. The photon bunching in a BS satisfying Eq. (6) follows coherence optics. Because the input photon phase relation of Eq. (7) can be satisfied by a BS, the anti-correlation scheme of Fig. 1 can also be implemented in Fig. 3. Figure 3(a) is a typical MZI scheme used for Young’s double-slit experiment for ${g}^{(1)}$ for one output measurements^11^, where the path length (phase) is controlled by the inserted phase shifter $\Psi$. Thus, the output fields in Fig. 3(a) are described by:

8 $$[\begin{array}{c}{E}_{3}\\ {E}_{4}\end{array}]=[BS][\Psi ][BS][\begin{array}{c}{E}_{0}\\ 0\end{array}]=\frac{1}{2}[\begin{array}{cc}(1-{e}^{i\psi }) & i(1+{e}^{i\psi })\\ i(1+{e}^{i\psi }) & -(1-{e}^{i\psi })\end{array}][\begin{array}{c}{E}_{0}\\ 0\end{array}],$$

where $[\Psi ]$ is a phase shifter matrix: $$[\Psi ]=[\begin{array}{cc}1 & 0\\ 0 & {e}^{i\psi }\end{array}]$$. According to MZI physics, the output determinacy is controlled by either $\psi \in \{0,\pi \}$ or the input channel of E_0_ (V or H): For details, see the Supplementary Information C. Here, the input channel selection or $\psi \in \{0,\pi \}$ in Fig. 3(a) is equivalent to choosing the sign of ${\varphi }_{n}$ in Eq. (7), determining which-way information in the output modes (see the swapping between dash-dot green curve and dashed blue curve for neighboring anti-correlations). The MZI determinacy (directionality) is due to destructive quantum interference, where the anti-correlation between two output fields indicates non-classical results^11^. If the two-photon bunching phenomenon on a BS is nonclassical^11^, then Fig. 3 satisfying Eq. (8) is also nonclassical (see two dots in Fig. 3(b)), violating Bell’s inequality (discussed *Discussion*). Thus, the anti-correlation $({g}^{(2)}=0)$ in a BS in Fig. 1 can be achieved in the MZI of Fig. 3(a), whether the input E_0_ is a single photon or coherent fields. The MZI of Fig. 3(a) used for anti-correlation is the second discovery of the present study.

## Discussion

In SPDC-HOM experiments, the phase relation of independent photon pair inputs in the time domain does not matter, as shown in Fig. 2. Moreover, there is a π/2$-$phase shift between the down-converted photons. These phase-matched input photon pairs have been overlooked in the quantum optics community, even though the input phase modulation has been observed^6^. The bunched output channel is deterministically decided based on the fixed input phase difference satisfying Eq. (7). On the contrary, random output in photon bunching can also be obtained by choosing a random input. As a result, an entanglement superposition can be achieved by combining output modes via two random input modes in ${\varphi }_{n}$ (see the two dots in Figs. 1(b) and 3(b))^6^: $|\Xi \rangle =\frac{1}{\sqrt{2}}(|{2}_{3}\rangle |{0}_{4}\rangle +|{0}_{3}\rangle |{2}_{4}\rangle )$ for single photons and $|{\rm{{\rm X}}}\rangle =\frac{1}{\sqrt{2}}({|{I}_{0}\rangle }_{3}{|0\rangle }_{4}+{|0\rangle }_{3}{|{I}_{0}\rangle }_{4})$ for coherent light. For a fixed input channel E_0_ in Fig. 3, this is accomplished by the random choice of $\psi \in \{0,\pi \}$. Although the coincidence detection for ${g}^{(2)}$ does not reveal which-way information of the bunched photons (fields), it is clear that the output mode strictly depends on the input phase choice. Thus, the symmetric sign of ${\varphi }_{n}$ plays a key role in entanglement superposition of the output modes satisfying Bell’s inequality. This result has never been discussed.

By adding the missing parameter ${\varphi }_{n}$ to E_1_ in Fig. 1(a), the following representations are found using Eqs. (4) and (5) for the outputs which satisfy anticorrelation:

9 $${E}_{3}=\frac{1}{\sqrt{2}}({E}_{1}+i{E}_{2})=\frac{1}{\sqrt{2}}(\pm i{E}_{0}+i{E}_{0})=\sqrt{2}i{E}_{0}\,{\rm{or}}\,0,$$

10 $${E}_{4}=\frac{i}{\sqrt{2}}({E}_{1}-i{E}_{2})=\frac{i}{\sqrt{2}}(\pm i{E}_{0}-i{E}_{0})=0\,{\rm{or}}\,\sqrt{2}{E}_{0}.$$

Both outputs in Eqs. (9) and (10) are strongly coupled together via the symmetric anti-correlation bases of Eq. (7), resulting in a nonclassical feature. This entanglement superposition composed of two nonclassical output modes has been separately discussed for unitary transformation in secured communications^23^, where the same analogy should suffice for $\psi \in \{0,\pi \}$ in Fig. 3. Brifely, the origin of the anti-correlation for photon bunching in HOM experiments satisfying nonclassicality^5^ or Bell’s inequality^3,4^ is destructive quantum superposition in a BS between the two input modes, where the missing parameter *φ*_*n*_ requires a particular phase relation between them. Bell’s inequality also suffices for the linear superposition of the anticorrelation modes determined by the missing parameter ${\varphi }_{n}$ with its symmetric property. As a result, the nonclassical feature can be accomplished coherently in an MZI. Furthermore, as seen in Fig. 3, Schrödinger’s cat may be achieved via superposition of the anti-correlation modes in coherence optics.

## Conclusion

Second-order anticorrelation between the two output modes of a beam splitter (or Young’s double-slit) was studied to understand its physical origins in the nonclassicality of photon bunching. Unlike the common understanding, which is limited to pure quantum optics of an anti-bunched nature governed by sub-Poisson statistics, the nonclassical phenomenon of photon bunching in HOM experiments was due to destructive quantum interference with a particular phase relationship satisfying coherence optics. Furthermore, an equivalent model of beam splitter-based anti-correlation using a typical MZI was presented and discussed. Finally, entanglement superposition of the output modes of an MZI was briefly discussed due to its potential application to unconditionally secured classical cryptography. The SPDC nonlinear process was analyzed for a HOM dip, in which individual input photon pairs governed by incoherence optics result in anti-correlation due to the pairs being phase-matched regardless of their frequency detuning. Moreover, the ${\rm{\pi }}/2$ phase shift required between the signal and idler photons for a HOM dip was explained to be a consequence in the SPDC process. Thus, the border-line between classical and quantum optics may be redrawn by coherence optics. Classicality cannot be limited by the wave nature of photons but by incoherence optics. In conclusion, coherence at a particular phase between two input photons is a necessary condition for the nonclassicality of anticorrelation, resulting in a HOM dip as well as Bell’s inequality. Thus, the present discovery, although seemingly in conflict with conventional single-photon-based quantum optics, opens the door to a new regime of quantum information compatible with coherence optics. The significance of this study is a better understanding of anticorrelation, where quantum superposition results in not only indistinguishability in the input modes but also no which-way information in the output entangled mode.

## Supplementary information

Supplementary information.
