# Supplementary material for: The origin of anticorrelation for photon bunching on a beam splitter
Source: Sci Rep. 2020 Apr 30;10:7309. doi: 10.1038/s41598-020-64441-2 (PMC7193647; doi:10.1038/s41598-020-64441-2)
Supplement: Supplementary file 1 — Supplementary information. [file 41598_2020_64441_MOESM1_ESM.docx]

**Supplementary Information**

The origin of anti-correlation for photon bunching on a beam splitter

B. S. Ham

1. **BS-based g^(2)^ calculations**

In Fig. 1, the phase matrix of $\left[ \Psi\right]$ is

$\left[ \Psi\right]=\left[ \begin{matrix} 1 & 0 \\ 0 & e^{i\psi} \end{matrix} \right]$. (A1)

After the phase shifter $\Psi$, the matrix of light fields $E_{3}^{'}$ and $E_{4}^{'}$ becomes:

$\left[ \begin{matrix} E_{3}^{'} \\ E_{4}^{'} \end{matrix} \right]=\left[ \Psi\right]\left[ \begin{matrix} E_{3} \\ E_{4} \end{matrix} \right]=\left[ \Psi\right]\left[ BS \right]\left[ \begin{matrix} E_{1} \\ E_{2} \end{matrix} \right]=\frac{1}{\sqrt{2}}\left[ \begin{matrix} 1 & 0 \\ 0 & e^{i\psi} \end{matrix} \right]\left[ \begin{matrix} 1 & i \\ i & 1 \end{matrix} \right]\left[ \begin{matrix} E_{1} \\ E_{2} \end{matrix} \right]=\frac{1}{\sqrt{2}}\left[ \begin{matrix} 1 & i \\ ie^{i\psi} & e^{i\psi} \end{matrix} \right]\left[ \begin{matrix} E_{1} \\ E_{2} \end{matrix} \right]$. (A2)

Thus, the MZI matrix becomes:

$\left[ \begin{matrix} E_{5} \\ E_{6} \end{matrix} \right]=\left[ BS \right]\left[ \Psi\right]\left[ BS \right]\left[ \begin{matrix} E_{1} \\ E_{2} \end{matrix} \right]=\frac{1}{2}\left[ \begin{matrix} \left( 1-e^{i\psi} \right) & i\left( 1+e^{i\psi} \right) \\ i\left( 1+e^{i\psi} \right) & -\left( 1-e^{i\psi} \right) \end{matrix} \right]\left[ \begin{matrix} E_{1} \\ E_{2} \end{matrix} \right]$. (A3)

The second-order interference in equation (6) is obtained by:

$g^{(2)}=\left\langle E_{3}E_{3}^{*}E_{4}E_{4}^{*} \right\rangle/\left( \left\langle E_{3}E_{3}^{*} \right\rangle\left\langle E_{4}E_{4}^{*} \right\rangle\right)$

$=\frac{1}{4I_{0}^{2}}\left\langle\left[ \left( E_{1}+iE_{2} \right)\left( E_{1}+iE_{2} \right)^{*}\left( {iE}_{1}+E_{2} \right)\left( {iE}_{1}+E_{2} \right)^{*} \right] \right\rangle$

$=\frac{1}{4I_{0}^{2}}\left\langle\left( E_{1}E_{1}^{*}-iE_{1}E_{2}^{*}+i\left( E_{1}E_{2}^{*} \right)^{*}+E_{2}E_{2}^{*} \right)\left( E_{1}E_{1}^{*}+iE_{1}E_{2}^{*}-i\left( E_{1}E_{2}^{*} \right)^{*}+E_{2}E_{2}^{*} \right) \right\rangle$

$=\frac{1}{4I_{0}^{2}}\left\langle{E_{1}E_{1}^{*}E_{1}E_{1}^{*}+E}_{1}E_{2}^{*}E_{1}E_{2}^{*}+\left( E_{1}E_{2}^{*} \right)^{*}\left( E_{1}E_{2}^{*} \right)^{*}+E_{2}E_{2}^{*}E_{2}E_{2}^{*} \right\rangle$

$+\left\langle iE_{1}E_{1}^{*}E_{1}E_{2}^{*}-iE_{1}E_{1}^{*}\left( E_{1}E_{2}^{*} \right)^{*}+E_{1}E_{1}^{*}E_{2}E_{2}^{*} \right\rangle$

$-\left\langle iE_{1}E_{2}^{*}E_{1}E_{1}^{*}-E_{1}E_{2}^{*}\left( E_{1}E_{2}^{*} \right)^{*}-iE_{1}E_{2}^{*}E_{2}E_{2}^{*} \right\rangle$

$+\left\langle i\left( E_{1}E_{2}^{*} \right)^{*}E_{1}E_{1}^{*}-\left( E_{1}E_{2}^{*} \right)^{*}E_{1}E_{2}^{*}+i\left( E_{1}E_{2}^{*} \right)^{*}E_{2}E_{2}^{*} \right\rangle$

$+\left\langle E_{2}E_{2}^{*}E_{1}E_{1}^{*}+iE_{2}E_{2}^{*}E_{1}E_{2}^{*}-iE_{2}E_{2}^{*}\left( E_{1}E_{2}^{*} \right)^{*} \right\rangle$. (A4)

1. For the incoherent inputs with different frequencies satisfying a random phase, the second-order interference results in:

$g^{(2)}=\frac{1}{4}\left\langle(1+e^{i(\Delta+\varphi)}e^{i(\Delta+\varphi)}+e^{-i(\Delta+\varphi)}e^{-i(\Delta+\varphi)}+1) \right.$

+(${ie}^{i(\Delta+\varphi)}-{ie}^{-i(\Delta+\varphi)}+1)$

+($-{ie}^{i(\Delta+\varphi)}-e^{i(\Delta+\varphi)}e^{-i(\Delta+\varphi)}-ie^{i(\Delta+\varphi)})$

+$({ie}^{-i(\Delta+\varphi)}-e^{-i(\Delta+\varphi)}e^{i(\Delta+\varphi)}+ie^{-i(\Delta+\varphi)})$

+$\left. (1+{ie}^{i(\Delta+\varphi)}-ie^{-i(\Delta+\varphi)}) \right\rangle$

$=\frac{1}{2}\left\langle1+cos2(\Delta+\varphi) \right\rangle$

$=\frac{1}{2}$, (A5)

where $\varphi=\varphi_{1}-\varphi_{2}$, $\Delta=\left( k_{1}-k_{2} \right)r-\left( w_{1}-w_{2} \right)t$, $E_{1}=E_{0}e^{i(k_{1}r-w_{1}t+\varphi_{1})}$, and $E_{2}=E_{0}e^{i(k_{2}r-w_{2}t+\varphi_{2})}$. Here, the difference frequency Δ contributes to coherence washout due to fast oscillation during the detector interrogation time.

1. For the coherent inputs with the same frequency $(\Delta=0)$, the second-order interference results in:

$g^{(2)}=\frac{1}{4}\left\langle(1+e^{i\varphi}e^{i\varphi}+e^{-i\varphi}e^{-i\varphi}+1) \right.$

+(${ie}^{i\varphi}-{ie}^{-i\varphi}+1)$

+($-{ie}^{i\varphi}-e^{i\varphi}e^{-i\varphi}-ie^{i\varphi})$

+$({ie}^{-i\varphi}-e^{-i\varphi}e^{i\varphi}+ie^{-i\varphi})$

+$\left. (1+{ie}^{i\varphi}-ie^{-i\varphi}) \right\rangle$

$=\frac{1}{2}\left( 1+cos2\varphi\right)$

$={cos}^{2}\varphi$. (A6)

1. Which-way information

Under the coherence optics for the same frequency inputs in Fig. 1(a), each output intensity I_j_ is denoted by:

$I_{3}=E_{3}E_{3}^{*}=\frac{1}{2}\left( E_{1}+iE_{2} \right)\left( E_{1}+iE_{2} \right)^{*}=(1+sin\varphi)$, (A-7)

$I_{4}=E_{4}E_{4}^{*}=\frac{1}{2}\left( {iE}_{1}+E_{2} \right)\left( iE_{1}+E_{2} \right)^{*}=(1-sin\varphi)$. (A-8)

As shown in Fig. A1, which way information is decided by the input phase difference $\varphi$. As an example two dots are denoted for $\varphi=\frac{\pi}{2}$, indicating the bunched photons (fields) directed to $E_{3}=2E_{0}$, while $E_{4}=0$. For $\varphi=-\frac{\pi}{2}$, however, the which way information in outputs is reversed, keeping the same anti-corrrelation.

Fig. A1. Which way information. The green dotted curve is $g^{\left( 2 \right)}(\tau=0)$.

1. **HBT**

In Fig. 1, for $E_{2}=0$, the output fields are described by:

$\left[ \begin{matrix} E_{3} \\ E_{4} \end{matrix} \right]=\left[ BS \right]\left[ \begin{matrix} E_{1} \\ 0 \end{matrix} \right]=\frac{1}{\sqrt{2}}\left[ \begin{matrix} 1 & i \\ i & 1 \end{matrix} \right]\left[ \begin{matrix} E_{1} \\ 0 \end{matrix} \right]$. (B-1)

Thus, $E_{3}=\frac{1}{\sqrt{2}}E_{1}$ and $E_{4}=\frac{i}{\sqrt{2}}E_{1}$. The second-order correlation for coincidence detection is thus:

$g^{\left( 2 \right)}\left( \tau\right)=\frac{\left\langle I_{3}(t)I_{4}(t+\tau) \right\rangle}{\left\langle I_{3}(t) \right\rangle\left\langle I_{4}(t) \right\rangle}=1$, (B-2)

where ${I_{3}=E}_{3}^{*}(t)E_{3}(t)$ and ${I_{4}=E}_{4}^{*}(t)E_{4}(t)$. Because the stellar light is chaotic and its line shape is Gaussian, each light-intensity used for the correlation measurement is also Gaussian, where the background value of $g^{(2)}$ is always one regardless of coincidence. As a result $g^{\left( 2 \right)}(\tau=0)$ becomes 2 in HBT, satisfying the following formula:

$g^{(2)}=1+\left| g^{(1)} \right|^{2}$, (B-3)

where $g^{(1)}$ is the first-order correlation or simply visibility satisfying $0\leq g^{(1)}\leq1$: see C. Foellmi, “Intensity interferometry and the second-order correlation function g(2) in astrophysics,” Astro. & Astrophys. **507**, 1719-1727 (2009); $g^{\left( 1 \right)}(\tau)=\frac{\left\langle E_{3}^{*}(t)E_{4}(t+\tau) \right\rangle}{\left\langle E_{3}^{*}(t) \right\rangle\left\langle E_{4}(t) \right\rangle}$.

1. **MZI directionality**

In Fig. 3, the output photons are determined by the input channel of E_0_. When E_0_ is incident vertically as shown, the output fields are obtained by equation (8):

$E_{3V}=\frac{1}{2}\left( 1-e^{i\psi} \right)E_{0}$, (C-1)

$E_{4V}=\frac{i}{2}\left( 1+e^{i\psi} \right)E_{0}$. (C-2)

The related intensities become:

$I_{3V}=\frac{I_{0}}{2}\left( 1-cos\psi\right)$, (C-3)

$I_{4V}=\frac{I_{0}}{2}\left( 1+cos\psi\right)$. (C-4)

Depending on $\psi\in\left\{ 0,\pi\right\}$, the output field is unidirectional, i.e., $I_{3V}=0; I_{4V}=I_{0}$ for $\psi=0$.

If the E_0_ channel is switched from vertical (V) to horizontal (H), then the output fields are:

$E_{3H}=\frac{i}{2}\left( 1+e^{i\psi} \right)E_{0}$, (C-5)

$E_{4H}=-\frac{1}{2}\left( 1-e^{i\psi} \right)E_{0}$. (C-6)

The related intensities become:

$I_{3H}=\frac{I_{0}}{2}\left( 1+cos\psi\right)$, (C-7)

$I_{4H}=\frac{I_{0}}{2}\left( 1-cos\psi\right)$. (C-8)

Compared with equations (C-3) and (C-4), the choice of E_0_ also swaps the output directionality. Therefore, the output fields are bunched, and its direction depends on the E_0_ channel or $\psi\in\left\{ 0,\pi\right\}$ (see Fig. 3(b)). Figure C1 is for horizon E_0_, representing swapping the bunched output.

Fig. C1. (a) A schematic of a coherence-optics-based HOM setup. BS, beam splitter; E_i_, i^th^ coherent light field; D_i_, i^th^ photodetector; M, mirror. (b) Numerical calculations for (a) for $\tau=0$. Red is for $g^{\left( 2 \right)}$ correlation (normalilzed). The blue (green) dotted curve is for I_3_ (I_4_) for $\psi=0$. At $\psi=\pm2n\pi$ the output is bunched into E_3_, while into E_4_ at $\psi=\pm(2n-1)\pi$.
